# Graph-generative neural network for EEG-based epileptic seizure detection via discovery of dynamic brain functional connectivity

**DOI:** 10.1038/s41598-022-23656-1

**Published:** 2022-11-08

**Authors:** Zhengdao Li, Kai Hwang, Keqin Li, Jie Wu, Tongkai Ji

**Affiliations:** 1 The School of Data Science, Chinese University of Hong Kong, Shenzhen, China; 2 Shenzhen Institute for Artificial Intelligence and Robotics for Society (AIRS), Shenzhen, China; 3 Department of Computer Science, State University of New York, New Paltz, NY USA; 4 Department of Computer and Information Sciences, Temple University, Philadelphia, PA USA; 5 Research Institute of Cloud Computing, Chinese Academy of Sciences, Dongguan, China

**Keywords:** Epilepsy, Electroencephalography - EEG, Machine learning

## Abstract

Dynamic complexity in brain functional connectivity has hindered the effective use of signal processing or machine learning methods to diagnose neurological disorders such as epilepsy. This paper proposed a new graph-generative neural network (GGN) model for the dynamic discovery of brain functional connectivity via deep analysis of scalp electroencephalogram (EEG) signals recorded from various regions of a patient’s scalp. Brain functional connectivity graphs are generated for the extraction of spatial–temporal resolution of various onset epilepsy seizure patterns. Our supervised GGN model was substantiated by seizure detection and classification experiments. We train the GGN model using a clinically proven dataset of over 3047 epileptic seizure cases. The GGN model achieved a 91% accuracy in classifying seven types of epileptic seizure attacks, which outperformed the 65%, 74%, and 82% accuracy in using the *convolutional neural network* (CNN), *graph neural networks* (GNN), and transformer models, respectively. We present the GGN model architecture and operational steps to assist neuroscientists or brain specialists in using dynamic functional connectivity information to detect neurological disorders. Furthermore, we suggest to merge our spatial–temporal graph generator design in upgrading the conventional CNN and GNN models with dynamic convolutional kernels for accuracy enhancement.

## Introduction

Neurological disorders are one of the leading causes of disability reported globally^1^. This is a severe healthcare problem awaiting effective solutions and treatment plans. For example, epileptic seizures cause 50 million people to suffer periodically, Alzheimer's disease (AD) affects 60 million patients facing dementia problems, and over 25 million people are suffering from schizophrenia globally, affecting all works of life with emotional depression, physical disability, and death threats^2–4^. These neurological disorders are related to complex functional connectivity and can be diagnosed by scalp electroencephalography (EEG) signals^5–9^.

EEG signal is characterized by specific frequency bands in onset occurrence. It contains rich spatial–temporal information on brain activities for discovering dynamic functional connectivity among various brain regions, identifying patterns of brain disorders, and localizing lesions such as epileptic foci^10–15^. In this paper, we focus on epileptic seizures, while our method can also be extended to other neurological disorders diagnosed by EEG.

Identifying EEG signals associated with different seizure attack types requires strenuous and time-consuming efforts by neurologists. It is highly desired to automate the process by ML methods. Moreover, due to the poor spatial resolution of scalp EEG recordings, it has been a challenge to identify abnormal regional or global connectivity by non-invasive scalp EEG procedure compared to intracranial electroencephalography (iEEG)^16^. Recent advance in machine learning has raised the hope that new models could help elevate the accuracy of scalp EEG-based diagnosis of neural circuit abnormalities, including epilepsy^17–21^.

A vast number of signal processing methods have been proposed for feature extraction in time-domain, frequency domain, time–frequency domain, or combinations^22–31^. Most of these methods require handcrafting hyperparameters, which is time-consuming and highly dependent on empirical experience. For example, the choice of channels, the number of levels in the flexible analytical wavelet transform (FAWT)^31^, tunable-Q wavelet transform TQWT^26^ or other wavelet transform-based methods, and the design of intrinsic mode functions (IMFs) in empirical mode decomposition (EMD)^30,32^, etc. Moreover, the selection or ranking of features extracted by signal processing methods requires empirical tuning since the choice of classifiers is affected by it^33^. Third, some signal processing methods are susceptible to variations across an increasing patient population or increasing channels^34,35^.

Deep learning methods can help to address those limitations by the highly non-linear universal approximation ability for automatic channel selection and feature extraction. The mainstream models such as *fully connected neural networks* (FCNN)^18,36^, *recurrent neural networks* (RNN)^37^, and *convolutional neural networks* (CNN)^38–42^ have demonstrated a great potential for seizure detection and classification by taking EEG signals as time-series data or a multi-channel image^43^. Liu, et al.^44^ proposed a hybrid model composed of CNN and RNN to extract spatial–temporal information. This model is enhanced with a bilinear pooling to exploit the second-order features from CNN and RNN. Ahmedt-Aristizabal, et al.^45^ designed a NMNs model with trainable neural plasticity combined with RNNs to obtain a dynamic strategy to store long-term temporal information and capture temporal relationships. However, these pre-trained models are inadequate to extract dynamic spatial information of the EEG signals, which is crucial to characterize the transient nature of the brain functional connectivity issues hidden in different regions of the brain lobes. These methods require manually-designed time windows and feature engineering to capture the dynamic spatial information. This feature engineering is very time-consuming and unstable to handle large datasets.

Other neural network models have been suggested, including graph neural networks (GNNs) for extracting deep spatial features on seizure detection tasks^46–52^. These GNN models are based on fixed neural connectivity topologies such as distance-based, which failed to capture the spatial dynamics associated with seizure attacks. The latest works utilizing transformer models^53^ have achieved some success in detecting connectivity thanks to the attention mechanism. However, it is still limited to discover new connectivity at different times.

In addition to detection or classification tasks, brain functional connectivity has been a very hot research topic for many years^54^. Revealing accurate connectivity within very small-time windows is critical to modeling the brain activity associated with neurological disorders. Although conventional methods such as functional magnetic resonance imaging (fMRI) can detect the changes of connectivity, the underlying dynamical mechanism of disruptive brain activity of the epileptic patient is still an open problem via scalp EEG. Discovering hidden non-linear mechanisms requires much more powerful machine learning or deep learning methods.

We developed a new class of graph-generative networks (GGN) to capture spatial and temporal features for high-resolution dynamic functional connectivity discovery that advances accurate seizure detection. We illustrate the new method with a clinically recorded epilepsy dataset provided by the Temple University Hospital^55^. This dataset contains seven types, i.e., *non-specific focal seizure* (FN), *generalized non-specific seizure* (GN), *simple partial seizure* (SP), *complex partial seizure* (CP), *absence seizure* (AB), *tonic seizure* (TN), and *tonic–clinic seizure* (TC).

Our testing results complement other detection methods based on fMRI and clinical factors^54,56,57^. The GGN approach helps brain specialists to detect early seizure attacks with more accurate results. Here, we report mainly the scientific results obtained from using four neural networks (CNN, GNN, GGN, and transformer) to detect epileptic seizure cases. We report relative performance and will elaborate on how to extend our GGN model to deal with other brain diseases in the “Discussions” section.

The main contributions of our works lie on three aspects. First, the hidden dynamic functional connectivity of the brain can be revealed by simply feeding scalp EEG signals into the proposed graph-generative model. This provides a powerful tool for analyzing the dynamic non-linearity of functional connectivity. Second, the GGN with the graph-generative technique yields higher automated detection accuracy and upgrades the medical efficiency in making personalized treatment plans for epilepsy patients. Finally, we developed a new adaptive and attentive graph convolution capability of GGN to amend the shortcomings or the low accuracy problem existing in CNN, GNN, and transformer models for seizure detection. This new capability of GGN outperforms the competing neural networks by a factor of 8.4–26.6% to reach a detection accuracy of 91%.

## Results

First, we introduce the main idea of using deep learning neural network for solving the seizure detection problem. Then we specify the GGN detection/classification framework. Experimental results are presented over a clinical seizure dataset involving 3,047 patient cases. Performance metrics are defined, and numerical results are presented with association maps, connectivity graphs, confusion matrices, classification clusters, and AUC-ROC curves. Then we illustrate the use of the generated functional connectivity graphs to achieve detection and classification purposes. We will discuss how to extend the work for connectivity discovery of other brain diseases based on scalp-EEG and elaborate on more methodological details of GGN model in the last sections.

### Framework of the automated seizure classification system

**Figure 1:**
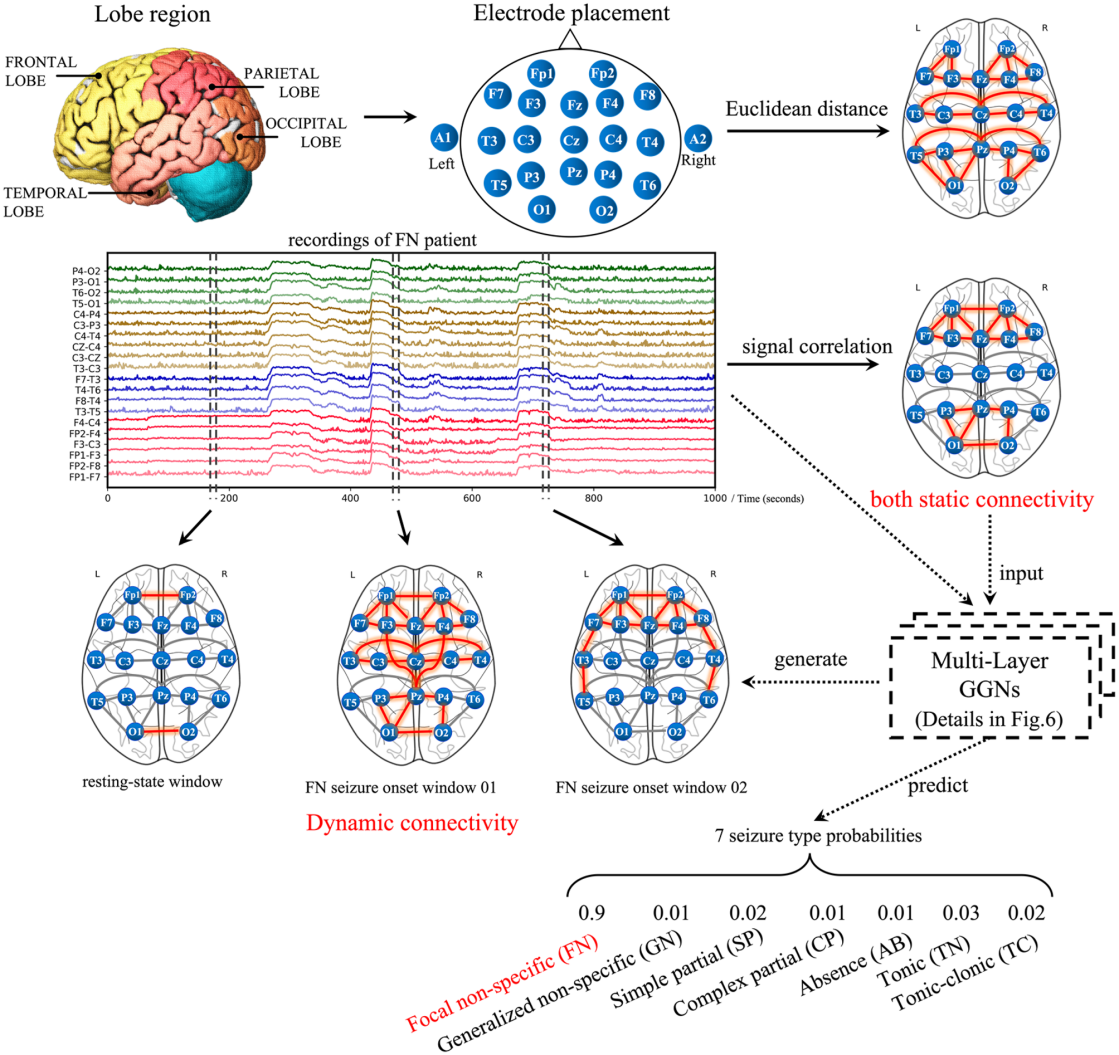
The key concept of scalp EEG-based framework for automated detection of dynamic functional connectivity during the onset of epileptic seizure attacks. The functional connectivity among lobe regions is indicated by the connection patterns among the electrodes. These connection patterns may vary all the time. The detection is based on generating connectivity graphs and onset attack classification. The newly introduced graph-generative neural network (GGN) plays a central role here. Details of GGN are given in Fig. 6 in the “Methods” section.

Our deep learning epileptic seizure classification system is illustrated in Fig. 1. We show how the EEG brainwave data are collected from 22 electrodes placed on the patient’s scalp. These electrodes record the EEG waveforms generated from different lobe regions of the human brain, i.e., frontal lobe, parietal lobe, occipital lobe, and temporal lobe. The electrode placement and the time-series recordings have rich information and non-linear spatial–temporal correlations among electrodes. Our GGN utilizes both spatial and temporal features for generating non-linear functional connectivity at different windows.

In general, the spatial features are extracted by the Euclidean distance of electrode placement or Pearson’s correlation among time-series signal from each electrode. The spatial features reflect the associations of each electrode and reveal the functional connectivity among the lobe regions. The edge connection strength represents the localized brain connectome. Figure 1 shows most local connections within the lobe regions. The connectivity induced by correlation-based methods is computed by a long-term window and only reflects linear relations. Nevertheless, these two static methods cannot handle dynamic and non-linear functional connectivity.

In Fig. 1, the GGN generates functional connectivity of a Focal non-specific patient over several small-time windows during the resting-state and seizure onset. At the resting-state, almost no significant connection is observed. While in onset window 01 and window 02, there exist two different connection patterns mainly in the Frontal lobe respectively. These two connection patterns locate in different regions not only in the frontal lobe and parietal lobe as in the correlated connectivity. The connections are cross-lobe with non-linearly association which indicates the high dynamics of functional connectivity. GGN models the connectivity in a non-linear way by using multi-layer generative neural networks with a universal approximation expressiveness.

### Spatial–temporal feature extraction

Our GGN detects dynamic functional connectivity among brain regions during seizure onset time. The connectivity graph generator learns the connectivity dynamics in the training process. These inter-lobe connectivity patterns are generated stochastically based on the learned distribution by GGN to reflect the dynamics in the EEG waveforms. EEG signals are recorded by the electrodes connected to the lobe position of the brain cerebrum. We need to measure both static and dynamic brain spatial features. Time-series signals need to be transformed to the frequency domain for extracting temporal features. The complex correlation between spatial and temporal features needs to be extracted simultaneously. The purpose is to reveal the functional connectivity along the seizure onset. Usually, the time-domain signal is transformed to band-filtered frequencies. Figure 1 shows the gamma band frequency of the EEG recordings.

### Abnormal connectivity and association maps

**Figure 2:**
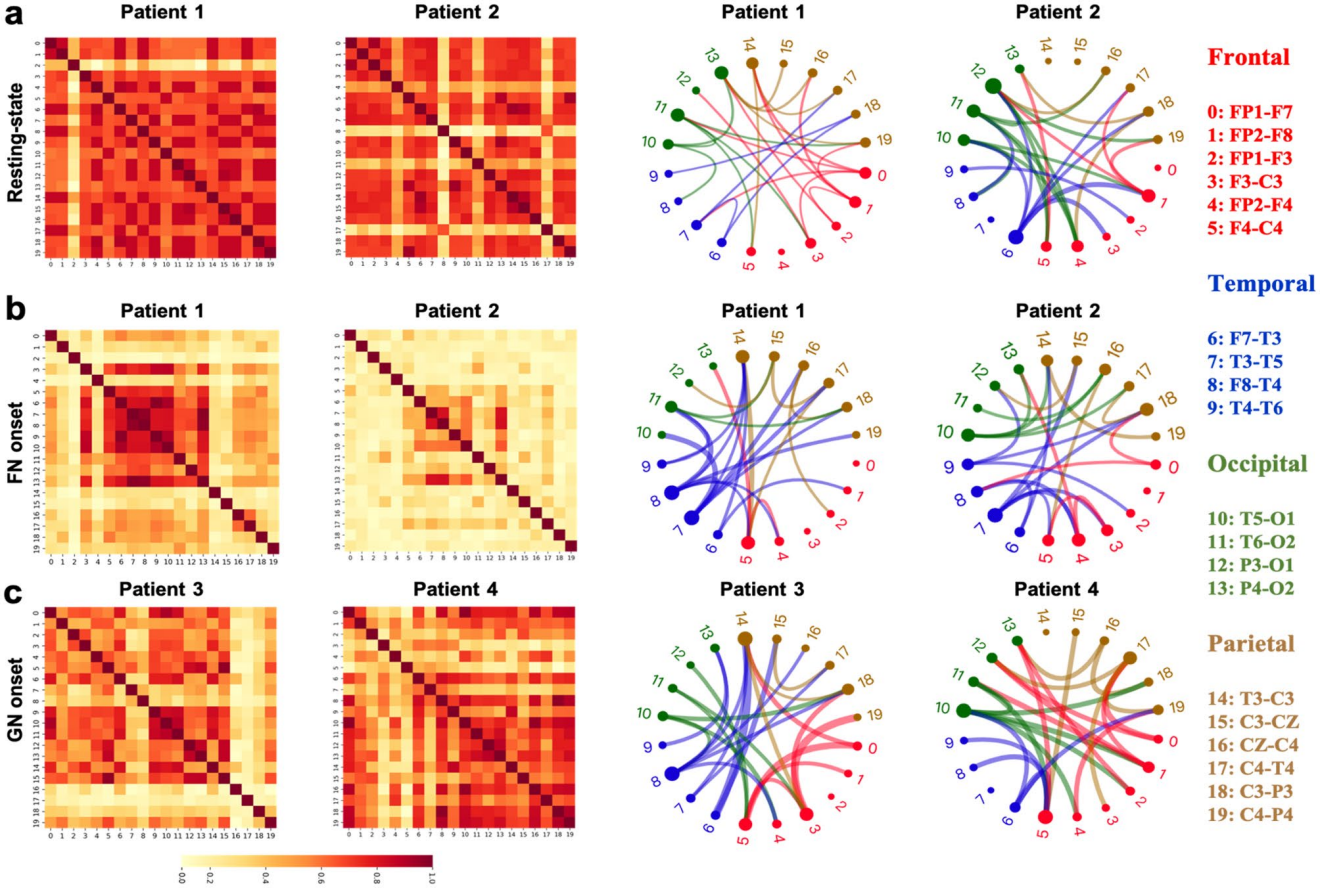
Visualization of normal and abnormal functional connectivity among four brain lobes identified by different colors and connection edge widths in the association maps and connectivity graphs. The association maps of the resting state, FN onset, and GN onset are shown on the left diagrams for four different seizure patients. The corresponding functional connectivity graphs are shown on the right circular diagrams. **(a).** The resting state of patient 1 and patient 2 with no disruptions in connectivity graphs. **(b).** The FN onset attack on patient 1 and patient 2. **(c).** GN onset of patient 3 and patient 4.

The association maps as the baseline are shown in the first two columns in Fig. 2, where each map is sorted by four main lobe regions. The FN attack is the most seizure attack type triggered by mainly local excitations. The GN seizure foci spread globally in various brain regions. Their functional connectivity graphs and association maps are shown in Fig. 2b and Fig. 2c. In Fig. 2, the pairwise associations are represented by *mutual information coefficient* (MIC)^58^ scores in the range {0,1} for 20 × 20 = 400 squares. The association MIC scores are shown by variable colors from dark brown to light yellow. The larger score corresponds to a higher association between the node pair. There was no significant abnormal activity detected in the normal case. The FN attack causes more excitation signals locally within the same brain lobe or affects only some nearby lobes.

### Clinically recorded seizure dataset

Our seizure dataset of EEG signals was recorded by Temple University Hospital^55^. We use the corpus released in May 2020, which contains 3047 seizure sessions (cases) at a sampling rate of 250 Hz. More details of seizure dataset are summarized in Table 1. For each training and testing round, we randomly shuffled 2033 cases for model training and the remaining 1014 cases for testing. We have compared the GGN testing results with other baseline ML/DL detection methods, including SVM^59,60^, CNNs based on Asif, et al.^38^, GNNs based on Tang, et al.^50^, and the transformer model by Yan, et al.^53^.

**Table 1:** Seizure dataset of 3047 epilepsy patient cases from Temple University hospital^55^. (Note that we ignore the MY type due to the small number of cases).

Seizure types	# Patients	# Cases
Focal non-specific (FN)	150	1836
Generalized non-specific (GN)	81	583
Simple partial (SP)	3	52
Complex partial (CP)	41	367
Absence (AB)	12	99
Tonic (TN)	3	62
Tonic–clonic (TC)	14	48
Myoclonic (MY)	3	2

### Functional connectivity graphs

We focus on functional connectivity discovery using non-invasive scalp-EEG signals. In Fig. 2, we present the detected abnormalities in functional connectivity within the same lobe or between different brain regions, and the association map generated by MIC. The normal and abnormal connectivity results are visualized by observing the changes in the edge connection patterns and signal strength on all connection edges among the frontal, temporal, occipital, and parietal brain lobes.

**Figure 3:**
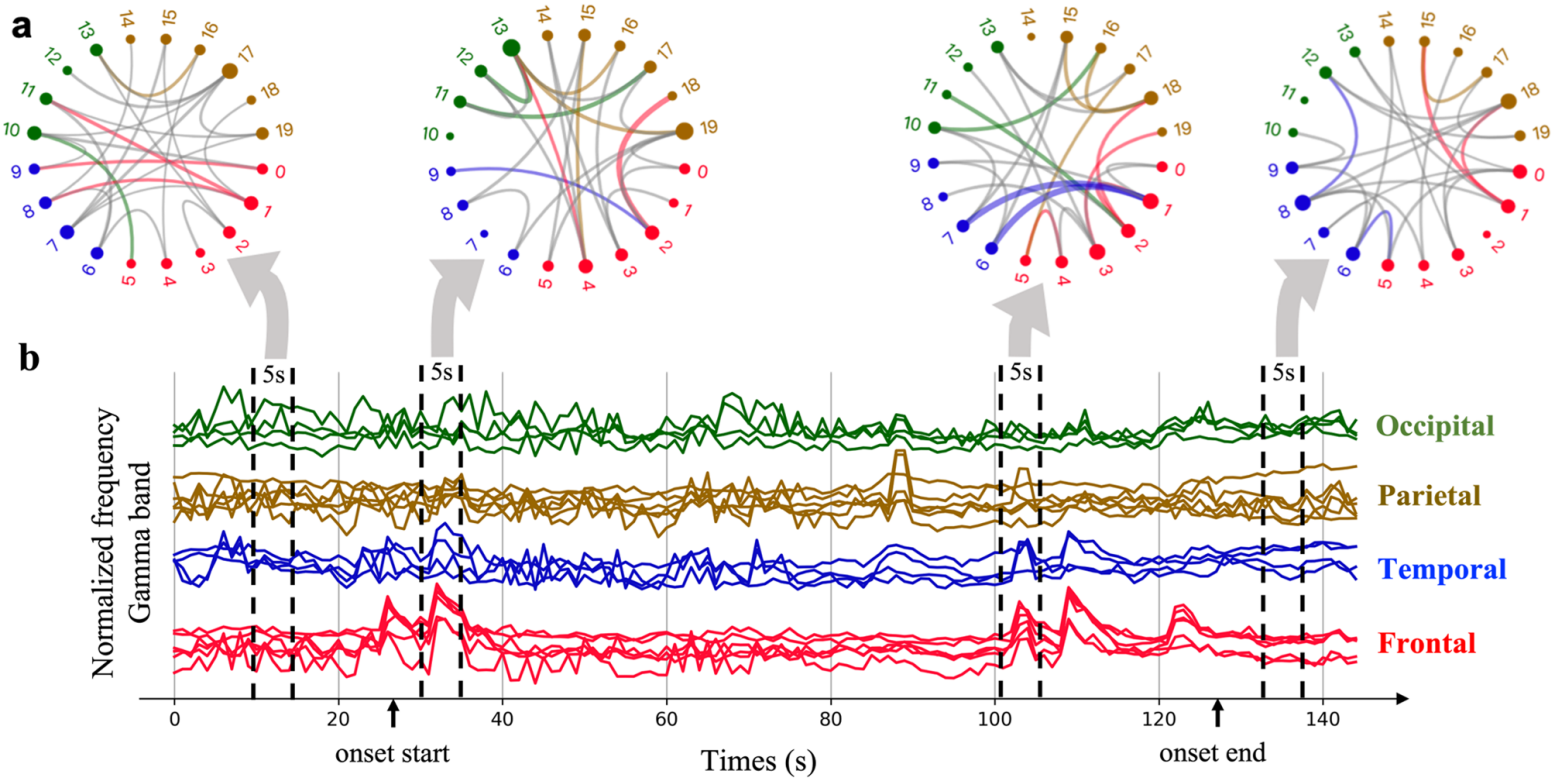
Spatial–temporal functional connectivity detected by GGN of an FN seizure patient onset. The light gray connection edges are measured at windows 1 and 4 before and after the seizure attack. Heavy colored edges in windows 2 and 4 are during the attack onset period**. (a).** The GGN captures connectivity changes in four stages by a 5s window from the resting state to the end of the seizure onset. The gray connection edge corresponds to connection strength below the measurement threshold. The colored edge connections are all exceeding the measured threshold with various excitation degrees. (**b).** Normalized brain waveforms in the gamma frequency band, where four observing windows were measured with a connectivity graph in (**a**). These observing windows cover 20 EEG electrodes from the seizure onset start to the onset ending in 150s.

Brain activity is complex, involving a lot of transient events. It is impossible to playback or validate. The association map is widely used to reflect the brain region correlations in a long term. Compared to the association map, our connectivity graph has a much higher resolution in detecting the changes in very short time windows. The width of the connection line reflects the probability that the two nodes have an interactive relationship that is crucial for the identification of different seizure types. The connection probability *p*_*ij*_ between channel *i* and channel *j* is calculated by our GGNs. Each connection of a connectivity graph is generated by calculating the expectation of samples that are drawn from this *p*_*ij*_. More details of this generation procedure can be found in “Methods” section. To visualize the functional connectivity, we define a connection strength based on this *p*_*ij*_ as follows:

1 $$\text{Connection Strength}(i,j) = {\text{exp}}\left( {\frac{{{\text{exp}}\left( {{\varepsilon *}p_{ij} } \right) - 1}}{{\upsigma }}} \right)$$

where the $\upvarepsilon$ and $\upsigma$ are two positive hyperparameters to control the strength range that is from the minimal value 1 to the maximal value $\mathrm{exp}\left(\frac{\mathrm{exp}(\upvarepsilon )-1}{\upsigma }\right )$. This definition is designed to reflect the exponential chaoticity of the epileptic EEG signals. Note that, in Fig. 2 and Fig. 3, we only show the top 25 weighted connections. In Fig. 3, we only color the edges with the strength greater than a hyperparameter β that has a significant strength gap compared with the rest edges which are in gray. More settings of these hyperparameters can be found in the supplementary material.

### Spatial–temporal connectivity

GGN can detect transitions in functional connectivity in different stages of seizure onset. This dynamic connectivity we call spatial–temporal connectivity. Compared to the association map such as Pearson correlation or MIC, which can only detect linear interaction relations in a fixed large time window, GGN can detect much more accurately by using a small-time window.

Figure 3 illustrates the spatial–temporal connectivity detected by GGN of one patient in an FN onset, where the onset started at 31s and ended at 128s. Figure 3b shows the dynamics of the frequencies in the gamma-band of 20 electrodes colored in four main lobes. We used a 5-s window to detect the connectivity shown in Fig. 3a. Links are colored if the connection strength exceeds a certain threshold.

The first connection pattern was generated before the seizure onset in the window scan from 10 to 15s. In this connection graph, only five connections are colored, which means most of the nodes are not associated with each other. The second connectivity graph was obtained at the beginning of the seizure onset at 31s. All signal curves are disturbed simultaneously, especially the frontal curves. The connectivity graph detected that fluctuation (a bold red link from the frontal lobe to the occipital lobe). The third connectivity graph was generated from using window 3 at 101s. Here, more colored links are observed.

There are two blue links from the temporal lobe to the frontal lobe, indicating a stronger correlation between the two lobe regions. The last connectivity graph was obtained after the seizure onset. Here, most edges are expressed by lightweight connections in gray color, similar to the connection pattern observed at the first window during the resting state. The two seizure strikes during windows 2 and 3 are contributed most by the waveform spikes at the frontal lobe.

### Confusion matrices and detection results

**Figure 4:**
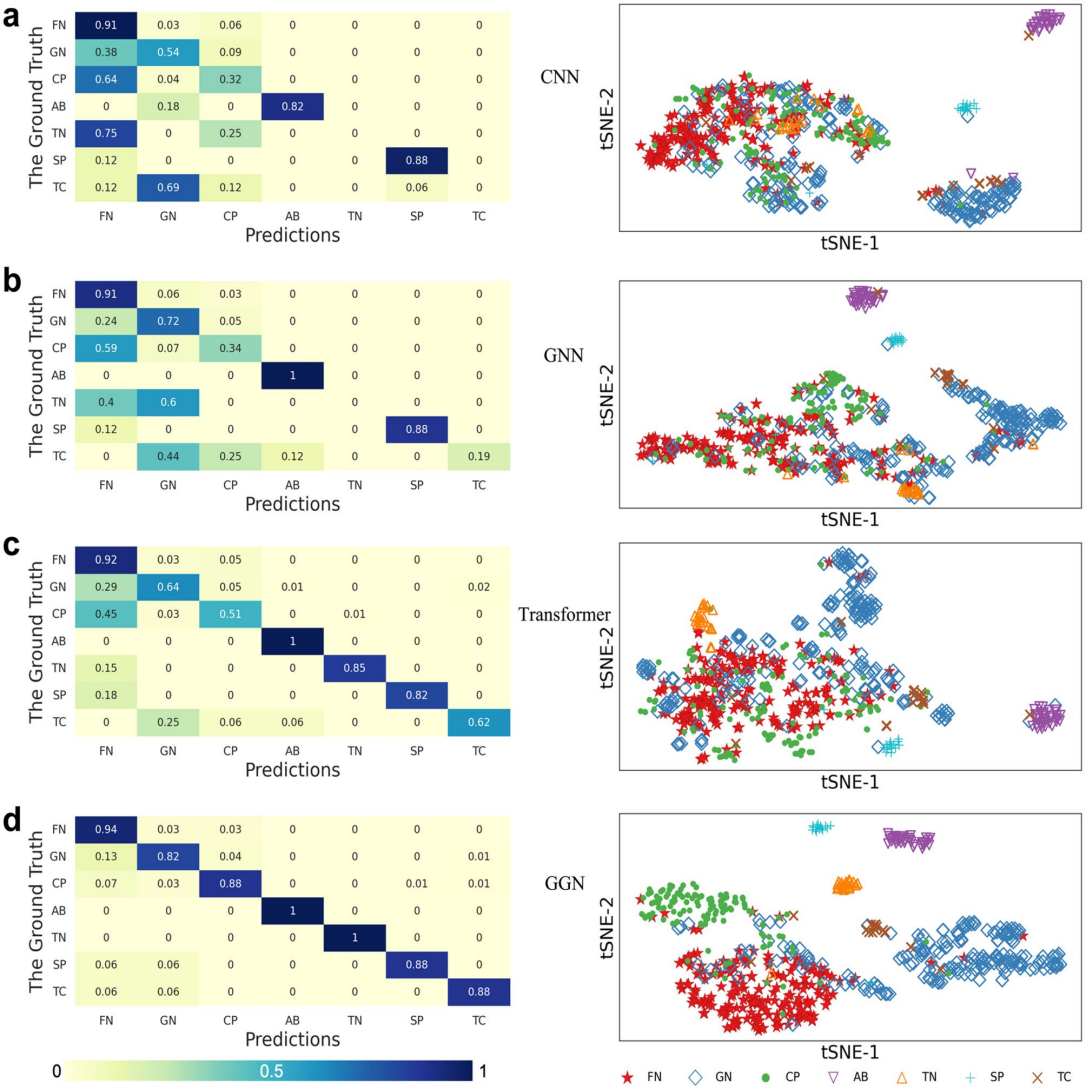
Seizure detection results represented by confusion matrices on the left side. The seizure classification clusters are shown on the right-hand 2-dimensional tSNE diagram using four distinct neural network detection models. **(a).** CNN shows many misclassified cases with many confused results due to the static convolution kernels used. **(b).** The GNN performs equally worst as CNN due to static graphs applied. **(c).** The transformer approach results in fewer confusions than CNN and GNN due to some encoder and decoder applied. **(d).** The GGN shows very little confusions with high accuracy along the main diagonal.

Figure 4 shows the confusion matrices and corresponding learned representation maps of the four DL methods for the classification of seven types of seizure attack. The results are given in Parts (a), (b), (c), and (d) for using the CNN, GNN, transformer, and our GGN models, respectively. The ground truth is shown on the y-axis and the predicted results on the x-axis. The number inside the boxes shows the detection accuracy. The relative magnitudes of the accuracy results are shown by different colored boxes. They are ranked from darker (higher accuracy) to light (lower accuracy) cases. The clustered results shown on the right are based on testing over 1014 seizure attack cases. Confusion matrix and 2-D clustering results do match each other closely in the four seizure detection systems.

In general, better detection accuracy is highlighted by the situation that most darker boxes appear in the principal diagonal (meaning no confusion at all) with zero or a few lighter boxes appearing in the off-diagonal entries. (meaning the existence of pairwise confusions). The reason why CNN did not perform well (Fig. 4a) is due to the fact that only static convolution kernels were used, which cannot capture the dynamic spatial features. The off-diagonal confusions reveal this weakness of CNN.

In Fig. 4b, we find that GNN performs equally worst as the CNN, due to the shortcomings of using fixed small filtering kernels. The transformer approach results in fewer confusions than CNN and GNN due to the attention mechanism and much higher feature dimensions, as shown in Fig. 4c. All AB attacks are well detected by four models without confusions. The FN, CP, and TN attack types have more confused results than the AB attack. The confusion mainly occurs in the first column. Other attack types could be misclassified as the FN type. The Transformer model has less confusion than that in CNNs and GNNs. Clearly, these detection results proved that the GGN outperforms all three seizure detection methods significantly.

### The t-SNE representation

To visualize the detection results, we use the t-SNE representation developed by Maaten and Hinton^61^. This is a dimension reduction method to map the high-dimensional data into a two-dimensional feature space. We introduce two dominating composite features: tSNE1 and tSNE2 in the 2D diagram shown in Fig. 4. Different attack types detected are represented by different icons with different colors. The distance between two attack points implies their similarity, where the shorter distance means higher similarity.

The t-SNE dimension reduction method produces a 2D representation map to approximate the probability ${q}_{ij}$ of the similarity between feature vectors of channel *i* and channel *j*. The right side of Fig. 4(a,b) shows heavy overlapping in sample locations on the 2D map. This implies many confusions exist in some seizure types. Both CNN and GNN are ineffective in separating the attack types FN, TN, and CP as they are heavily jammed in the same map areas. Compared to CNN, the GNN has less overlapped coverage in the jammed areas.

GNN has less equally bad as the CNN, due to using fixed filtering kernels. The transformer approach results in fewer confusions than CNN and GNN due to the attention mechanism and higher dimensions shown in Fig. 4c. The Transformer is not effective in detecting the FN and GN seizure attack types. However, the transformer shows perfect detection 100% on the AB attack. The GGN model has little confusions on the main diagonal in Fig. 4d.

The transformer-based method has resulted in even fewer overlapped coverages. Finally, we observe that the GGN model can separate different attack types almost perfectly in Fig. 4d. Almost all attack types are separable. In summary, these four seizure detection methods are ranked as GGN, transformer, GNN, and CNN from high to low in terms of detection accuracy. The testing of 1,014 validation cases has proved this relative performance ranking results.

### ROC performance of 4 competing seizure detection methods

**Figure 5:**
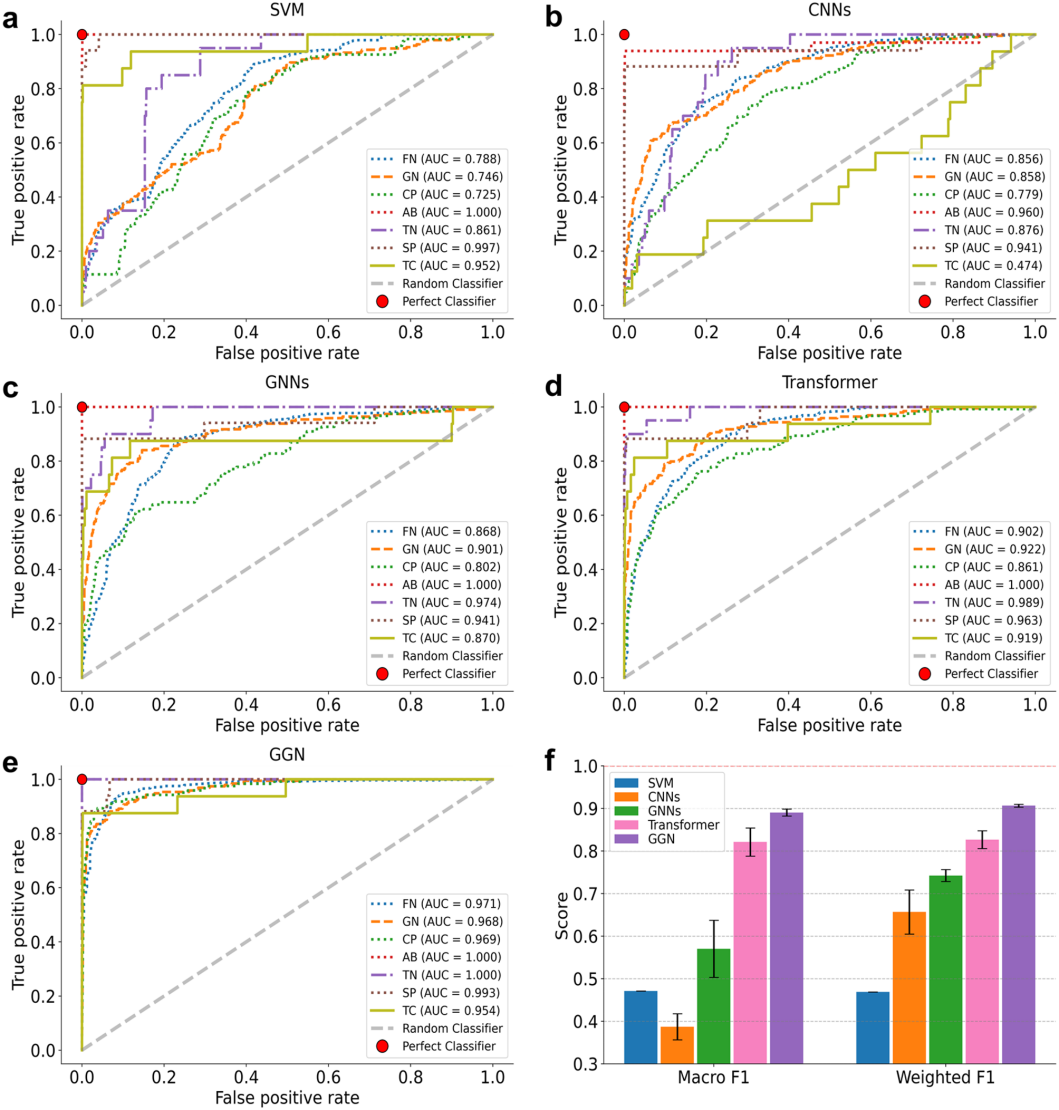
Classification results are expressed as AUC-ROC curves for seven seizure attack types. **(a).** Results of the SVM model. **(b).** Results by using the CNN model. **(c).** Results by using the GNN model. **(d).** Transformer detection results. **(e).** Results by the GGN model. **(f).** Accuracy scores of two compound performance metrics for five distinct machine learning and neural network models for the classification of epileptic seizure attack types.

The *receiver operating characteristic curve* (ROC) for the seven seizure attack types is shown in Fig. 5. The ROC curve considers the trade-off between *true-positive rate* and *false-positive rate* of the detection process. The area under the ROC curve (AUC) is the probability that a classifier can distinguish a positive and a negative correctly. The larger the area, the higher the detection accuracy. Using the SVM method in Fig. 5a, the FN, GN, and CP attacks have low performance. The CNN has the lowest detection performance in Fig. 5b. The TC attack experiences steady improvement as we switch from CNN to GNN, transformer, and GGN models in Fig. 5(b–d).

The AB attack type achieves the perfect score instantly at zero FNR. In the ideal case (a single red solid dot), the TPR stays at 100% with a zero FNR score. A random classifier follows the diagonal gray line. In fact, all seven seizure detection results have ROC curves between the ideal case and random classifier. Figure 5b shows that the CNN has a worse performance closer to the random classifier.

The GNN results shown in Fig. 5c improve to some extent by moving towards the ideal case. The transformer improves further, as seen in Fig. 5d. The GGN results surpass all other four detection models with a fast-saturating range near the upper left corner of the ROC space. The number inside the boxes shows the detection accuracy. The relative magnitudes of the accuracy results are shown by different colored boxes. They are ranked from darker (higher accuracy) to light (lower accuracy) cases.

### Compound performance metrics

Due to the severe imbalance of seizure types among K seizure types, we consider the following three metrics to obtain a comprehensive and fair comparison. Let ${TP}_{i}$ be the average true-positive rate, ${FP}_{i}$ be the average false-positive rate, and ${FN}_{i}$ be the average false negative rate. We apply the detection rate of type *i* seizure attack as follow:

2 $${\text{F}}1 = \frac{{2{\text{TP}}_{i} }}{{2{\text{TP}}_{i} + {\text{FP}}_{i} + {\text{FN}}_{i} }}$$

3 $${\text{Macro F}}1 = \frac{1}{{\text{K}}}\mathop \sum \limits_{{{\text{k}} = 1}}^{{\text{K}}} {\text{F}}1_{k}$$

4 $${\text{Weighted F}}1 = \mathop \sum \limits_{{{\text{k}} = 1}}^{{\text{K}}} {\upomega }_{k} {\text{F}}1_{k}$$

Now we are ready to show two *compound performance metrics* as follows: *Macro F1* (Eq. 3) and *Weighted F1* (Eq. 4) scores for all *K* attack types. The Weighted F1 is under the proportion ${w}_{i}$ of the *i-th* seizure type. The F1 score is applied to define the average or weighted harmonic mean of the precision and recall and represents the overall performance^60,62^. In Fig. 5f. we compare the relative performance of the five seizure detection methods evaluated by the two above metrics on 1014 testing samples. Macro F1 and weighted F1 scores report the performance of *K* = 7 seizure attack types. Our GGN can achieve an average or weighted detection rate (or the score label in Fig. 5f between 88 and 91%, which is rather impressive.

The transformer approach achieves an 83% performance. The GNN scheme ranked the third place. These three methods are in the same trend on both two metrics. In summary, the GGN scheme achieved a 91% detection score, compared with 65%, 74%, and 82% accuracy using CNN, GNN, and transformer models, respectively. For the weighted F1 performance, the CNN model achieves a low 66% accuracy. The Macro F1 performance is relatively poor (36%) due to the sharp disparity in the attack types. The SVM has a low 46% under both compound performance measures. Based on these measured results, we recommend using the weighted F1 compound performance to amend the disparity situations in the data set applied.

The imbalanced class distribution usually downgrades the performance. A model extracting more aspects of features can alleviate this problem. The CNN-based models have been very successful in static image understanding applications. But CNN fails to extract global spatial information in the seizure detection problem. This is because CNN with fixed small convolution kernels that fails to capture the global connectivity.

Our GNN applies a wide and flexible range of kernels to enable the construction of more reliable connectivity graphs to make an accurate classification. Although the transformer models have achieved great success on NLP and even image tasks, they are still limited in detecting complex topological changes.

## Discussions

In general, our DL method can assist brain specialists or neuroscientists with more concrete evidence to monitor or assess patient conditions more accurately. Our GGN can be applied to detect not only epileptic seizure attacks, but also other neurological disorders driven by complex spatial–temporal scalp EEG waveform features. The extended work may include the detection of Alzheimer’s disease (AD) or the determination of the patient’s severity of schizophrenia symptoms, etc. Moreover, our graph-generative method provides a higher resolution and efficient tool for detecting the changes in brain functional connectivity via scalp EEG.

EEG analysis of AD is usually based on some statistical signal processing methods, such as phase leg index and minimum spanning trees. These methods assumed that the EEG signals could distinguish from other dementia diseases in several frequency bands and specific lobe areas^63^. The functional connectivity of AD patients shows the presence of the small-world network features by graph theory^56^. These features can be well detected by our GGN model, as an even smaller local abnormality of FN seizure has been well detected.

The functional connectivity of schizophrenia has shown strong correlations between the cortical oscillatory dynamics and schizophrenic syndrome by analysis of the band-filter EEG signals^64^. The beta and gamma bands (> 20 Hz) have much higher correlations. The schizophrenia is similar to the GN epileptic seizure attack. To detect the long-range oscillatory dynamics, one needs to use a longer time window. The latent functional connectivity depends on patient specificity. The connectivity generator in GGN can support researchers in detecting abnormal functional connectivity in EEG signals with higher spatial resolution without supervised connectivity labels. The larger public clinical datasets may demonstrate even higher performance in using the GGN model.

Compared with the CNN-based model^38^ and the transformer-based model^53^ in our experiments, our GGN model uses much shallower hidden layers. This implies that we use fewer learnable parameters and thus has lower computational complexity. The computational complexity of GNN-based model^50^ is close to ours. Compared to other signal processing methods and most conventional ML methods such as SVM, PCA, etc.^65^, the GGN model takes longer time on training, while the prediction time of the pretrained model is usually very short depending on the computing server used. In this regard, our GGN model takes longer overhead time to yield satisfactory results than that of most signal processing methods.

In practice, the CNN can achieve a considerably high accuracy by additional preprocessing of raw data into images. For instance, Asif, et al.^38^ have pointed out that pre-processing the raw EEG signal by generating a set of saliency-encoded spectrogram maps with different scales can significantly improve the performance. This multi-saliency information alleviates the limitation of the convolution kernels to some extent by involving more global information. Due to the fixed topology such as the correlation-based graph, conventional GNN-based models face the same problem. Thus, merging our connectivity generator design will be able to upgrade the performance in extracting dynamic spatial information. The connectivity graphs can be applied directly in GNN to alleviate the limitation of using a fixed topology.

The main limitations of our GGN model are identified in two aspects. The first weak point lies in the fact we handle a small fixed number of EEG channels during the training process. This will limit our pre-trained model to fixed EEG recording settings. Secondly, our sampling procedure requires repeated iterations in the prediction step, which may take longer time to yield higher accuracy. This procedure may affect the real-time applications when the iteration number is large. In the future, we will try to fix these two limitations to widen clinical applications in other neurological disorders based on EEG signal analysis.

Our contributions are identified in three scientific aspects: Firstly, we provide a machine learning framework for brain specialists to efficiently identify epileptic seizure attacks. This AI approach can reinforce neurological diagnosis to reach some unbiased method overcomes this difficulty with increased accuracy.

Secondly, our graph-generative neural network approach offers the first step to revealing the abnormality in brain functional connectivity. Our GGN work can cope with the dynamics involved in the spatial and temporal feature extraction process. We have amended the shortcomings in using the CNN, GNN, and transformer models in solving the seizure detection problem without well pre-selected features and channels.

Thirdly, our work can be applied by neuroscientists to study other neurological diseases in the brain functional connectivity analysis, such as Alzheimer's disease and schizophrenia, etc., because an accurate analysis is hindered by the low-resolution of non-invasive scalp EEG. The proposed graph-generative method facilitates the accurate discovery of functional connectivity with small observation windows.

## Methods

Detecting brain connectivity is still a wide-open problem mostly studied in graph theory or statistical methods such as the correlations of time-series data. The new GGN model generates the connectivity graphs from scalp EEG waveform signals. We reveal the changes in brain functional connectivity within the same brain lobe or across multiple lobes by simply feeding the scalp EEG signals to the GGN. This plays a critical role in the detection and diagnosis of various seizure attack types. We present the holistic GGN model, and its key components, including connectivity graph generator and attentive convolution mechanisms to enable seizure detection and classification. Then we elaborate the procedure of the seizure classification and connectivity graph generation. Finally, we discuss the GGN model training settings and the cross-validation testing process.

### The GGN architecture

**Figure 6:**
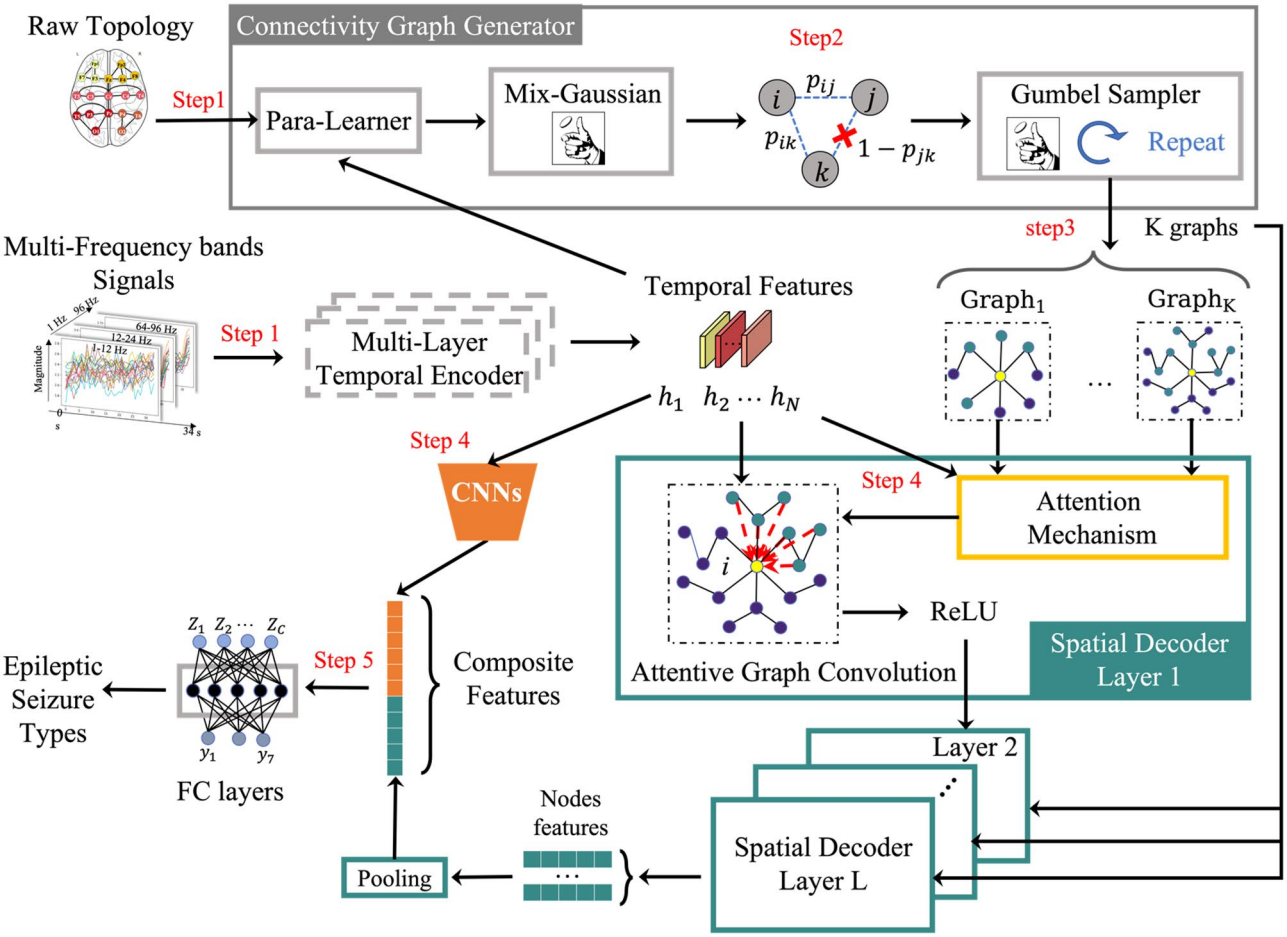
Major functional components in the graph-generative neural network (GGN). The connectivity graph generator at the top box; a multi-layer spatial decoder (green box) with attentive graph convolutional supported by attention mechanism; shallow CNNs (in orange color); fully connected classifier. Dynamic latent graph generation applies raw topology data measured by the EEG probes. The spatial decoder is built with attentive graph convolution layers in concatenation with the use of CNN hidden layers for the final classification of the seizure attack types. Steps 1 to 3 outline the major steps in discovering brain functional connectivity.

Figure 6 illustrates the holistic architecture of GGN containing main functional modules in different colors for seizure classification and two detailed functional modules, i.e., connectivity graph generator and spatial decoder. To explore the functional connectivity that reflects how brain regions interact with each other when epileptic seizure attacks. The connectivity graph generator models the connectivity as a random variable that varies over time. Different seizures are related to specific seizure onset regions. This generator is trained during the process of classification training.

The *temporal encoder* learns the temporal features of EEG data. It takes the time-series data as a 3-D tensor in ${R}^{N\times {C}_{in}\times T}$, where *N* is the number of electrodes, termed as channel size, ${C}_{in}$ is the feature dimension, and *T* is the time step length.  Output feature dimension is denoted as ${C}_{out}$. The *spatial encode*r extracts spatial features and decodes the final seizure representation for classification. The *output classifier* is a two-layer fully-connected neural network with ReLU non-linear function^66^. The fully-connected (FC) classifier maps the composite features to the probability of seizure attack type attached with a softmax function.

### Connectivity graph generator and spatial decoder

Here, we elaborate on two key functional modules, i.e., *connectivity graph generator* in gray box and the *spatial decoder* in green box on the bottom side of Fig. 6.

The *connectivity graph generator* is composed of three sequentially stacked components as shown in Fig. 6 gray box, i.e., Para-Learner, Mix-Gaussian module, and Gumbel-Sampler. The Para-Learner contains three independent GNNs known as *message-passing neural networks*. It takes a raw topology and temporal features as input. Each independent GNN learns some parameters of a mixture Gaussian distribution separately^68^. Since the epileptic networks vary over time, leading to disruptions in brain functional connectivity, modeling the connectivity from a probabilistic perspective can be more accurate than a deterministic approach. The Mix-Gaussian module generates the probabilistic distribution of each connection of two nodes. Consider two electrode nodes *i* and *j.* The ${p}_{ij}$ denotes the probability of an edge connecting the node *i* and node *j*. The Gumbel-Sampler generates all edges of a graph based on the distribution from Mix-Gaussian. The parameters are updated via the *stochastic gradient descent* (SGD) scheme by using a Gumbel-softmax trick^67^ which can make the sampling procedure differentiable.

The *spatial decoder* is a multi-layer structure that is composed of several attentive graph convolutions in each layer. The spatial decoder extracts spatial information and generates distinguished node-level representations that are fed into a pooling layer for classification. The attentive graph convolution is aligned with latent graphs from the graph generator, and is operated by a novel attention mechanism to refine the latent graphs. The output of the last spatial decoder layer L is all *N* nodes representations.

### Objective function applied

The raw data set is collected from brainwave signals on 20 electrode EEG channels. The training dataset is represented by {$\mathcal{X},\mathcal{Y},{A}^{(0)}$}, where the $\mathcal{X}$ consists of *N* = 2,033 time-series samples, $\mathcal{Y}$ is the corresponding labels, and ${A}^{\left(0\right)}$ is the initial connection weights among the electrodes. Let *s* be the sample index in the training dataset. Let ${\varvec{\theta}}$ be the collective set of all learnable parameters in our  model. The *likelihood function* for the optimization goal of our GGN framework is defined below:

5 $$\begin{array}{*{20}c} { {\mathcal{L}}\left( {{\varvec{\theta}};\left\{ {{\mathcal{X}},{\mathcal{Y}},A^{\left( 0 \right)} } \right\}} \right) = \mathop \prod \limits_{{{\text{s}} = 1}}^{N} \mathop \sum \limits_{{{\text{m}} = 0}}^{M} P_{{\varvec{\theta}}} \left( {{\mathcal{Y}}_{s} , A^{\left( m \right)} {|} {\mathcal{X}}_{s} ,A^{\left( 0 \right)} } \right)} \\ \end{array}$$

Given the training dataset, our purpose is to determine the model parameters ${\varvec{\theta}}$ to yield the maximum likelihood function $\mathcal{L}$ of the GGN detection outcomes. The difficulty lies in the fixed initial connectivity ${A}^{\left(0\right)}$ that did not exploit the spatial information. Thus, it cannot predict any dynamic connectivity. Therefore, we need to generate a series of *M* latent connectivity graphs $\{ A^{\left( m \right)} \}_{{{\text{m}} = 1}}^{M}$.

### Attentive graph convolution

A novel attention mechanism refines the graphs ($\text{Grap}\text{h}_{1}$ to $\text{Grap}\text{h}_{\mathrm{K}}$) from the connectivity graph generator to generate local and global spatial representations. These graphs have various neighboring ranges. For instance, the $\text{Grap}\text{h}_{\mathrm{K}}$ has more green nodes than $\text{Grap}\text{h}_{\mathrm{1}}$ indicating a larger neighborhood range. Therefore, the attention mechanism is to automatically learn the best neighboring range from these graphs according to the different seizure events. The refined graph (with red arrows) then is fed into the graph convolution which aggregates all neighboring node features and transforms into a new feature space. As shown in the spatial decoder layer 1, the attentive graph convolution on the target node *i* (in yellow) of $\text{the graph}$ is to aggerate the features *h*_*i*_ of its neighbors (in green) to update its representation. The result of the attentive graph convolution is then fed into a ReLU and is taken as the input of the next layer.

### Procedure for seizure classification and connectivity generation

The onset attack classification is outlined in the following five steps, where the black arrow represents the data flow. Step 1 is to prepare the spatial and temporal input, i.e., the raw topology generated by distance-based and correlation-based connectivity and the frequency signals transformed from the time-domain signals. The temporal input is fed into the multi-layer temporal encoder (any invariants of RNNs) to extract temporal features in a 3-D tensor. These temporal features and the raw topology are fed into the connectivity graph generator to generate dynamic connectivity graphs shown in Step 3. The temporal features are also fed into the multi-layer spatial decoder and CNNs in Step 4. The multi-layer spatial decoder learns the node representations and fed into a pooling layer which sums all node representations as a graph-level 1D representation. The shallow CNNs takes the temporal features as 3D images, where the channel of temporal features is taken as the image channel. The output of the CNNs is also flattened to a 1D representation. The representations from the pooling layer and the CNNs are then concatenated into a composite representation for the classifier in Step 5. In our settings, we use a two-layer fully-connected neural network with ReLU as the classifier to predict the seizure attack types.

As shown in the gray box in Fig. 6, *connectivity graph generator* discovers dynamic functional connectivity. Below are the major steps of this connectivity graph generation process. First, we feed the raw topology and the temporal features of one patient from the temporal encoder into the Para-Learner, which generates K parameter sets of a mixture Gaussian distribution*.* Then we draw two samples from the mixture Gaussian for each pair of electrode nodes. These samples are used to generate the edge probability as shown in Step 2. The connection strength of the existing edge between node *i* and node *j* is recorded by ${p}_{ij}$. Then we draw a set of graphs by Gumbel-Sampler based the connection probabilities obtained in Step 3.

The expectation of these graphs is taken as the initial functional connectivity graphs. Due to the changes in connection dynamics, these graphs need to be updated in many layers. Finally, we feed the graphs from the Step 3 into the attentive graph convolution module. We designed a novel attention mechanism to automatically learn the importance of each connectivity graph, and generate the most contributed graph for the classification. Details of the attention mechanism can be found in the supplementary. We take the connectivity graphs obtained in the last attentive convolution layer for classification purposes and are supposed to be the final functional connectivity.

### Model training of the GGN

We use the *evidence lower bound (*ELBO)^68^ for the model training instead of the Eq. 5. Maximizing this lower bound is equivalent to minimizing the weighted cross-entropy (WCE)^69^ where the weights are the proportion of each type. We train our GGN using WCE as the loss function and apply the Adam^70^ as the SGD optimizer. The lower bound we derived is as follows:

6 $${\text{ELBO }} = \mathop \sum \limits_{{{\text{s}} = 1}}^{N} \mathop \sum \limits_{{{\text{m}} = 1}}^{{\text{M}}} Q_{{\varvec{\omega}}} \left[ {log\frac{{P_{{\varvec{\theta}}}^{{\left( {m,s} \right)}} }}{{Q_{{\varvec{\omega}}}^{{\left( {m,s} \right)}} }}} \right]{ }$$

where the two learnable probabilities ${P}_{{\varvec{\theta}}}^{(m, s)}$ and ${Q}_{{\varvec{\omega}}}^{(m, s)}$ are defined by ${P}_{{\varvec{\theta}}}^{(m, s)}=Prob_{\varvec{\theta}}\left({\mathcal{Y}}_{s}, {A}^{\left(m\right)}| {\mathcal{X}}_{s},{A}^{\left(0\right)}\right),$ and ${Q}_{{\varvec{\omega}}}^{(m, s)}=Pro{b}_{{\varvec{\omega}}}\left({A}^{\left(m\right)}| {\mathcal{X}}_{s},{A}^{\left(0\right)}\right)$. Here, the ${\varvec{\omega}}$ and ${\varvec{\theta}}$ are the learnable neural network parameters of the GGN. Specifically, ${\varvec{\omega}}$ is attributed to the connectivity graph generator, and ${\varvec{\theta}}$ is attributed to other modules of the GGN.

We designed an alternative training scheme that is similar to the expectation–maximization (EM) method^68^ to optimize parameters ${\varvec{\theta}}$ and ${\varvec{\omega}}$. The trained model is cross validated by testing the remaining 1014 sample data from the Temple University Hospital dataset. All experimental settings, training procedures, and derivation of the lower bound can be found in the supplementary materials on the posted website.

## Supplementary Information

Supplementary Information.

## Data Availability

The data used in this paper are released by the Temple University Hospital through the following website at. https://isip.piconepress.com/projects/tuh_eeg/. The size of the dataset could vary with the neurological disease types. We choose the typical size that can be managed efficiently in our local cloud servers.

## Code availability

Users must seek permission to use the code for non-commercial use from the lead author: zhengdaoli@link.cuhk.dedu.cn. Note that all brain images in Fig. 1 and Fig. 6 were created via the python package nilearn in version 0.9.1^71^. The source codes for our GGN and all re-implemented baseline models are available at the following web site at https://github.com/ICLab4DL/GGN. The official source code of the GNN-based method is available at https://github.com/tsy935/eeg-gnn-ssl. The Transformer-based code is available at: https://github.com/xutianyu540/Transformer-network-with-a-three-tower-structure. The GAT model for GNN code is available at: https://github.com/Diego999/pyGAT.
