# Supplementary material for: Graph-generative neural network for EEG-based epileptic seizure detection via discovery of dynamic brain functional connectivity
Source: Sci Rep. 2022 Nov 8;12:18998. doi: 10.1038/s41598-022-23656-1 (PMC9643358; doi:10.1038/s41598-022-23656-1)
Supplement: Supplementary file 1 — Supplementary Information. [file 41598_2022_23656_MOESM1_ESM.pdf]

Supplementary

EEG channel selection

The raw dataset TUSZ v1.5.2 [1] collected from the clinical scalp-EEG equipment, recording 22 channel signals from the international 10-20 system. We select 20 channels out of 22 and use TCP montage [2] as the time-series input signals, i.e., FP1-F7;F7-T3;T3-T5;T5-O1;FP2-F8;F8-T4;T4-T6;T6-O2;T3-C3;C3-CZ;CZ-C4;C4-T4;FP1-F3;F3-C3;C3-P3;P3-O1;FP2-F4;F4-C4;C4-P4;P4-O2.

Frequency feature extraction

Suppose the time-series signal of channel *i* is denoted as $\hat{X}_i = \{\hat{x}_i^t\}_{t=0}^{T-1}$ with in total *T* time steps. Then we compute the different frequencies in various bands of the raw signal in the one second time window by Fast Fourier Transform $\mathcal{F}(\cdot)$: $x_i^k = \mathcal{F}(\hat{X}_i) = \sum_{t=0}^{T-1} \hat{x}_i^t \, e^{-\frac{2\pi i}{T}kt}$ with real part kept only, and take the *log* of the amplitude: $X_i = \{log(Amplitude(x_i^k))\}_{k=0}^{T-1}$. To this end, a time-series sample is denoted as $\mathcal{X}_s = \{X_i\}_{i=1}^{W} \in \mathbb{R}^{W \times C_0 \times T}$, where the *W* is number of channels that equals to 20, C_0 is the total number of frequencies that equals to 244, and *T* is total seizure attacking time in second.

Construction of raw topology

We use $A^{(0)}$ (a weighted adjacency matrix) to denote the initial raw topology that is constructed by a Gaussian kernel [3] with a threshold applied on the Euclidean distance of each two electrode. The weight value A_{ij} in $A^{(0)}$ between the node i and j is calculated by:

$$A_{ij} = \begin{cases} exp\left(-\dfrac{|dist(i,\ j)|^2}{\sigma}\right) & if\ dist(i,j) < \gamma, \\ 0 & otherwise \end{cases} \tag{1}$$

Where the *dist(i, j)* represents the Euclidean distance between node *i* and node *j, and* σ, γ are two positive hyperparameters.

Derivation of the ELBO

We apply the *maximum likelihood estimation* (MLE) on the training dataset $\{\mathcal{X}, \mathcal{Y}, A^{(0)}\}$ with *N* samples, where the likelihood function $\mathcal{L}$ is defined as follows:

$$\mathcal{L}(\boldsymbol{\theta}; \mathcal{X}, \mathcal{Y}, A^{(0)}) = \prod_{s=1}^{N} P_{\boldsymbol{\theta}}(\mathcal{Y}_s | \mathcal{X}_s, A^{(0)}) \tag{2}$$

Where the $\boldsymbol{\theta}$ denotes the learnable parameters of GGN model $P_{\boldsymbol{\theta}}$. By introducing a set of latent connectivity graphs as the random variables $A^{(m)}$, the $P_{\boldsymbol{\theta}}(\mathcal{Y}_s | \mathcal{X}_s, A^{(0)})$ is the marginal distribution of $P_{\boldsymbol{\theta}}(\mathcal{Y}_s, A^{(m)} | \mathcal{X}_s, A^{(0)})$, so that $\mathcal{L}$ is equivalent to:

$$\mathcal{L}(\boldsymbol{\theta}; \{\mathcal{X}, \mathcal{Y}, A^{(0)}\}) = \prod_{s=1}^{N} \sum_{m=1}^{M} P_{\boldsymbol{\theta}}(\mathcal{Y}_s, A^{(m)} | \mathcal{X}_s, A^{(0)}) \tag{3}$$

Imposing the log likelihood and a variational inference on $\mathcal{L}$:

$$\begin{aligned}
&\log \mathcal{L}(\boldsymbol{\theta}; \mathcal{X}, \mathcal{Y}, A^{(0)}) \\
&= \log \prod_{s=1}^{N} \sum_{m=1}^{M} P_{\boldsymbol{\theta}}(\mathcal{Y}_s, A^{(m)} | \mathcal{X}_s, A^{(0)}) \\
&= \sum_{s=1}^{N} \log \sum_{m=1}^{M} P_{\boldsymbol{\theta}}(\mathcal{Y}_s, A^{(m)} | \mathcal{X}_s, A^{(0)}) \frac{Q_{\boldsymbol{\omega}}(A^{(m)} | \mathcal{X}_s, A^{(0)})}{Q_{\boldsymbol{\omega}}(A^{(m)} | \mathcal{X}_s, A^{(0)})} \\
&= \sum_{s=1}^{N} \log \sum_{m=1}^{M} Q_{\boldsymbol{\omega}}(A^{(m)} | \mathcal{X}_s, A^{(0)}) \frac{P_{\boldsymbol{\theta}}(\mathcal{Y}_s, A^{(m)} | \mathcal{X}_s, A^{(0)})}{Q_{\boldsymbol{\omega}}(A^{(m)} | \mathcal{X}_s, A^{(0)})} \\
&\leq \sum_{s=1}^{N} \sum_{m=1}^{M} Q_{\boldsymbol{\omega}}(A^{(m)} | \mathcal{X}_s, A^{(0)}) \, log \, \frac{P_{\boldsymbol{\theta}}(\mathcal{Y}_s, A^{(m)} | \mathcal{X}_s, A^{(0)})}{Q_{\boldsymbol{\omega}}(A^{(m)} | \mathcal{X}_s, A^{(0)})} \\
&= \text{ELBO}
\end{aligned}$$

Here, the $\boldsymbol{\omega}$ and $\boldsymbol{\theta}$ are the learnable neural network parameters of the GGN, specifically, $\boldsymbol{\omega}$ is attributed to the connectivity graph generator, and $\boldsymbol{\theta}$ is attributed to all the other modules of the GGN.

Gumbel Sampler

Denote a mixture Gaussian distribution as $\mathcal{M} = \sum_{k=1}^{K} \pi_k N_k(\mu_k, \sigma_k)$ with K components for each node, where the (π_k, μ_k, σ_k) are learned by a GNN-based model respectively. To approximate $Q_\omega(A_{i,j}^{(m)} = 1 | \mathcal{X}_s, A^{(0)})$, we first draw two samples S_i, S_j from $\mathcal{M}$, by using two reparameterization tricks:

$$O_\pi = one\_hot(\pi) = \text{Gumb}(\pi, \tau) \tag{4}$$

$$S_i = O_\pi^T \mu + O_\pi^T \sigma \cdot n_i \tag{5}$$

where $\pi = [\pi_1, \dots, \pi_K]^T$, and $\text{Gumb}(\cdot, \tau)$ is a Gumbel-softmax reparameterization trick [4] to get a continuous one-hot representation O_π of a categorical sample drawn from the distribution π, and a temperature hyperparamer τ is to control the smoothness of O_π. Based on O_π, sampling from $\mathcal{M}$ could be differentiable by the second reparameterization trick shown in equation (4), where $\mu = [\mu_1, \dots, \mu_K]^T$, $\sigma = [\sigma_1, \dots, \sigma_K]^T$, and n_i is a sample drawn from a standard normal distribution. Repeated equation (4) and (5), we get the other sample S_j. Then the probability of the connection between node i and node j is computed by:

$$Prob(A_{i,j}^{(m)} = 1 | S_i, S_j) = \text{Sigmoid}(S_i \cdot S_j) \tag{6}$$

After calculated all probabilities of all node pairs, we obtain the $A_s^{(m)}$ for the input sample $\mathcal{X}_s$. Each sample in a training batch $\mathcal{B}$ corresponds to a new $A_s^{(m)}$ by repeating the equation (4)-(6). Here, we take the expectation of the $A_s^{(m)}$, i.e., $A^m = \mathbb{E}_{s \sim \mathcal{B}}[A_s^{(m)}]$.

Attention mechanism and attentive graph convolution

A mixture Gaussian $\mathcal{M}_m$ corresponds to a latent connectivity graph $A^{(m)}$, the attention mechanism is to learn a dynamic weight α_m for each $A^{(m)}$ and take a weighted sum of all $A^{(m)}$ with its weight. Suppose $\boldsymbol{Z}^{L-1}$ is the temporal representation at layer $L-1$ for a single node from temporal encoder, the α_m is calculated by two new representations $\boldsymbol{V}$ and $\boldsymbol{Q}$ learned from $\boldsymbol{Z}^{L-1}$.

$$H_m = conv(\boldsymbol{Z}^{L-1}, A^{(m)}) \tag{7}$$

$$V_m = Linear(H_m) \tag{8}$$

$$Q = Linear(\boldsymbol{Z}^{L-1}) \tag{9}$$

Where the $conv(\cdot)$ is the graph convolution operation over the $A^{(m)}$ and $Linear(\cdot)$ is a linear transformation operation that maps the representation into a *S* dimension space. The composed matrix $\boldsymbol{V}$ is denoted as $\boldsymbol{V} = [V_1, \ldots V_M]$. Here, $\boldsymbol{V} \in \mathbb{R}^{S\times M}$, $Q \in \mathbb{R}^{S}$. Then each attention weight is calculated by:

$$[\alpha_1, \ldots, \alpha_M] = softmax\left(\frac{Q^{\boldsymbol{T}}\boldsymbol{V}}{\sqrt{S}}\right) \tag{10}$$

At this end, the *attentive graph convolution* at layer *L* is defined as follows:

$$\boldsymbol{Z}^{L} = ReLU\left(\sum_{m=1}^{M} \alpha_m H_m\right) \tag{11}$$

References

[1] Obeid, Iyad, and Joseph Picone. "The Temple University Hospital EEG Data Corpus." Frontiers in Neuroscience 10 (2016)

[2] Lopez, Silvia, Aaron Gross, Scott Yang, Meysam Golmohammadi, Iyad Obeid, and Joseph Picone. "An analysis of two common reference points for EEGs." In *IEEE Signal Processing in Medicine and Biology Symposium* (SPMB), pp. 1-5. IEEE, 2016.

[3] Shuman, David I., et al. "The emerging field of signal processing on graphs: Extending high-dimensional data analysis to networks and other irregular domains." *IEEE Signal Processing Magazine* 30.3 (2013): 83-98.

[4] Eric Jang, Shixiang Gu, and Ben Poole. Categorical reparameterization with gumbel softmax. In *International Conference on Learning Representations*, 2017.
